# Central Pain Processing in Early-Stage Parkinson's Disease: A Laser Pain fMRI Study

**DOI:** 10.1371/journal.pone.0164607

**Published:** 2016-10-24

**Authors:** Christine Petschow, Lukas Scheef, Sebastian Paus, Nadine Zimmermann, Hans H. Schild, Thomas Klockgether, Henning Boecker

**Affiliations:** 1 Functional Neuroimaging Group, Department of Radiology, University of Bonn, Bonn, Germany; 2 Department of Neurology, University of Bonn, Bonn, Germany; 3 Department of Radiology, University of Bonn, Bonn, Germany; Hospital General Dr. Manuel Gea Gonzalez, MEXICO

## Abstract

**Background & Objective:**

Pain is a common non-motor symptom in Parkinson’s disease. As dopaminergic dysfunction is suggested to affect intrinsic nociceptive processing, this study was designed to characterize laser-induced pain processing in early-stage Parkinson’s disease patients in the dopaminergic OFF state, using a multimodal experimental approach at behavioral, autonomic, imaging levels.

**Methods:**

13 right-handed early-stage Parkinson’s disease patients without cognitive or sensory impairment were investigated OFF medication, along with 13 age-matched healthy control subjects. Measurements included warmth perception thresholds, heat pain thresholds, and central pain processing with event-related functional magnetic resonance imaging (erfMRI) during laser-induced pain stimulation at lower (E = 440 mJ) and higher (E = 640 mJ) target energies. Additionally, electrodermal activity was characterized during delivery of 60 randomized pain stimuli ranging from 440 mJ to 640 mJ, along with evaluation of subjective pain ratings on a visual analogue scale.

**Results:**

No significant differences in warmth perception thresholds, heat pain thresholds, electrodermal activity and subjective pain ratings were found between Parkinson’s disease patients and controls, and erfMRI revealed a generally comparable activation pattern induced by laser-pain stimuli in brain areas belonging to the central pain matrix. However, relatively reduced deactivation was found in Parkinson’s disease patients in posterior regions of the default mode network, notably the precuneus and the posterior cingulate cortex.

**Conclusion:**

Our data during pain processing extend previous findings suggesting default mode network dysfunction in Parkinson’s disease. On the other hand, they argue against a genuine pain-specific processing abnormality in early-stage Parkinson’s disease. Future studies are now required using similar multimodal experimental designs to examine pain processing in more advanced stages of Parkinson’s disease.

## Introduction

Parkinson’s disease (PD) is a neurodegenerative disorder causing progressive deterioration of motor function [1]. So-called ‘non-motor symptoms’ [2–6] are nowadays increasingly recognized as integral components of PD, including cognitive [7], mood [8], autonomic [9], and sleep [10,11] disturbances. Although pain symptoms are less widely appreciated as non-motor manifestations of PD, already the original description of ‘the shaking palsy’ by James Parkinson identified pain as part of the clinical symptomatology, often occurring as one of the first signs at disease onset [12]. Pain in PD is under recognized and undertreated [13], affecting roughly 40–60% of PD patients [5,6,13–19], and is linked to depression, decreased quality of life [20], female gender, age, disease duration and severity [16]. According to Ford [14], pain in PD can be differentiated into: (i) musculoskeletal pain, (ii) radicular, or neuropathic pain, (iii) dystonia-related pain, (iv) akathitic discomfort, and (v) primary, or central parkinsonian pain. A study involving 176 home-living PD patients [13] found elevated pain levels on the ‘Bodily Pain Scale’, with musculoskeletal pain making up 70%, followed by dystonic pain (40%), radicular-neuropathic pain (20%), and central neuropathic pain (10%).

The concept of ‘primary parkinsonian pain’ independent of PD motor symptoms was initially outlined by Souques in 1921 describing bizarre stabbing and burning sensations [21]. It is understood as a primary dysfunction of central pain pathways and is attributed to central dopamine loss, as it occurs frequently in untreated patients or during “OFF” periods, and is modifiable by dopamine substitution [22]. Schestatsky et al. provided a neurophysiological characterization in nine PD patients with primary central pain, as compared to 9 PD patients without pain, and 9 healthy control subjects [23]. PD patients with primary central pain had lower heat pain and laser pinprick thresholds, higher laser-evoked pain amplitudes, and less habituation of the laser-induced sudomotor skin responses than PD patients without pain and control subjects [23]. Abnormalities in pain processing were attenuated after L-Dopa substitution [23], implicating ‘primary parkinsonian pain’ to be a direct consequence of the hypodopaminergic state in PD. Likewise, pain in PD can be provoked by dopamine agonist withdrawal [24].

The neuronal mechanisms mediating pain processing in PD are as yet not well understood. Functional neuroimaging has been relevant for delineating central mechanisms underlying motor [25–28] and cognitive [29–31] disturbances in PD. More recent imaging research has also focused on non-motor sensory aspects [32,33] of the pathophysiological spectrum (for review see: [34]). Central pain processing in PD has, however, exclusively been addressed by one previous positron emission tomography (PET) H_2_^15^O activation study. Brefel-Courbon et al. compared pain thresholds before and after administration of levodopa in nine PD patients and nine controls, along with cerebral activity during experimental nociceptive stimulation [35]. In the OFF, pain thresholds were significantly lower than in the control cohort, but levodopa medication raised these thresholds significantly. The OFF was associated with relatively increased pain-evoked activation of the insula, prefrontal cortex and anterior cingulate cortex, which was attenuated by L-dopa treatment. These examinations in a rather small cohort provided interesting first indications that L-dopa administration modulates pain responses and pain thresholds in PD [35]. A major limitation, however, is the very liberal level of significance (p < 0.01, uncorrected), questioning the general validity and reproducibility of the reported findings. Also, the authors did not test for the presence of Parkinson-associated neuropathies [36], which may affect the propagation of standardized nociceptive stimuli into the parkinsonian brain, hence, introducing variance of peripheral, rather than central origin.

Here, we intended to resolve the above mentioned ambiguities, (i) by excluding patients with polyneuropathy, (ii) by using event-related functional magnetic resonance imaging (fMRI) with its intrinsically higher spatial resolution compared to PET, (iii) by applying conservative statistical thresholds, (iv) by combining imaging with standardized heat pain threshold measurements, psychophysiological pain intensity ratings, and skin-conductance measurements. We focused exclusively on OFF state examinations, as we primarily conceived to trace potential abnormalities of central pain processing in PD patients, using a multilevel experimental approach.

## Methods

### Subjects

In a telephone call subjects were informed about study objective as well as procedure and were interviewed about exclusion criteria which were psychotropic substance abuse, medication affecting pain perception, psychiatric disorders, diabetes mellitus, and evidence of peripheral neuropathy. At a first visit interested subjects were informed about the study procedure again. After subjects gave a first written informed consent for pretests they underwent an electroneurographic polyneuropathy screening.

We screened a total of N = 83 persons (N = 43 PD; N = 40 healthy controls) for potential enrollment into the study and had to rule out four cases with preexisting claustrophobia, seven cases with severe psychiatric disorders, two cases with tinnitus, six cases with missing interest in finally participating in the study, and two cases with metallic implants. Of these, another 10 PD patients and 9 healthy controls had to be excluded because of peripheral neuropathy evidenced in the electrophysiological workup. 6 PD patients and 7 healthy controls withdrew participation during different recruitment stages.

**Fig 1:**
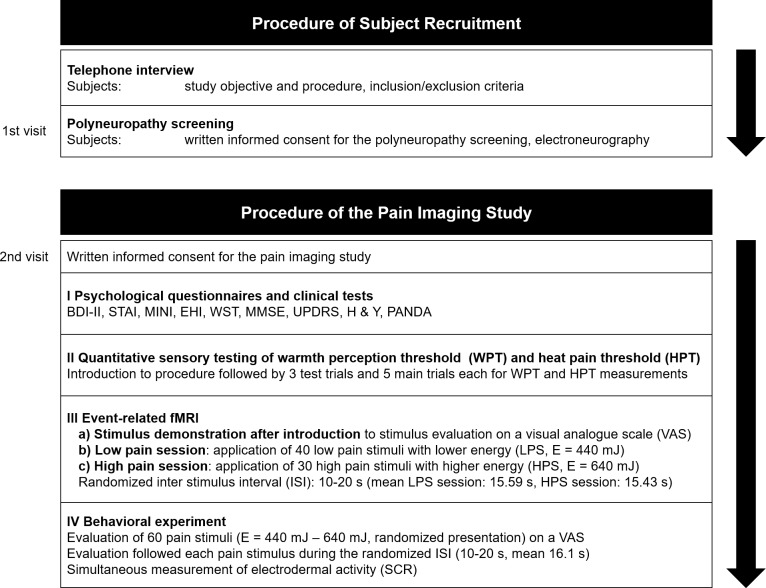
Procedure of subject recruitment and pain imaging study. Subject recruitment included a first telephone interview and was followed by a first visit for an electroneurographic polyneuropathy screening. At a second visit enrolled subjects underwent the experimental pain study, consisting of psychological questionnaires, clinical tests, quantitative sensory testing, event related fMRI, and behavioral pain testings.

After screening, 15 healthy controls (HC) and 15 patients with the clinical diagnosis of idiopathic PD according to the diagnostic criteria of the UK-Parkinson’s Diseases Society Brain-Bank [37] could be enrolled. All subjects gave a second written informed consent to the study examinations. One PD patient and one healthy control had to be excluded after enrollment in the MRI study due to claustrophobia (2). Incidental MRI findings were found in one further PD and one further HC, resulting in a total sample with complete and analyzable data of 13 HC (four female, nine male, mean age 54.46 ± 9.60 years) and 13 PD (four female, nine male, mean age 48.62 ± 5.85 years; for clinical details see Table 1). All subjects were right-handed [38]. At a second visit subjects took part in the following study procedure (Fig 1). Patients were studied after > 12 hours overnight treatment withdrawal.

**Table 1:** Clinical characterization of patients with early-stage idiopathic Parkinson’s disease.

Patient no.	Sex	Age	Years of disease duration	PANDA cognition	PANDA mood	UPDRS I	UPDRS II	UPDRS III	UPDRS IV	UPDRS total	HY stage	Pharmacological treatment
P01	m	44	7	24	1	3	9	10	3	25	1.5	LD, RGN, RPN
P02	m	53	8	26	0	2	9	13	1	25	1.5	AMN, LD, SGN, PPX
P03	m	45	5	30	2	0	14	11	2	27	1.0	RGN
P04	m	49	1	15	2	1	2	5	2	10	1.0	PPX, RGN
P05	m	44	4	26	0	0	5	12	0	17	1.0	AMN, PPX, RGN
P06	m	45	3	14	1	1	6	12	1	20	1.0	CD, EC, LD, PPX, RGN
P07	f	53	5	30	0	0	5	14	6	25	1.0	CD, LD, RGN, RPN
P08	m	42	5	27	3	1	13	21	4	39	2.0	CD, LD, RGN
P09	m	43	4	28	4	1	11	12	1	25	1.5	CD, AMN, LD, SGN,
P10	f	54	2	24	3	3	8	15	0	26	2.0	AMN, PPX, SGN
P11	f	44	6	24	3	0	4	22	1	27	1.0	CD, LD, BP
P12	m	57	5	29	3	2	4	15	0	21	1.0	LD, PPX, RGN
P13	f	59	3	26	1	0	2	11	0	13	1.0	CD, LD, RGN
M		48.62	4.46	24.85	1.77	1.08	7.08	13.31	1.62	23.08	1.27	
± SD		± 5.85	± 1.94	± 5.05	± 1.36	± 1.11	± 3.95	± 4.44	± 1.81	± 7.25	± 0.39	

Clinical test data were collected after at least 12 hours withdrawal of pharmacological antiparkinsonian treatment.

AMN: amantadine, BP: biperiden, CD: carbidopa, EC: entacapone, f: female, LD: L-dopa, M: mean, m: male, PANDA: Parkinson Neuropsychometric Dementia Assessment, PPX: pramipexole, RGN: rasagiline, RPN: ropinirole, SD: standard deviation, SGN: selegiline, UPDRS: Unified Parkinson’s Disease Rating Scale, HY stage: Hoehn & Yahr stage.

The study was approved by the local Ethics Committee of the University Hospital of Bonn, Germany (reference number 115/11). All participants gave written informed consent and were recruited between November 2012 and October 2014.

### Psychological questionnaires and clinical tests

Psychological and clinical tests included Beck Depression Inventory (BDI-II) [39], Mini International Neuropsychiatric Interview (MINI) [40], Mini-Mental State Examination (MMSE) [41], German vocabulary test (Wortschatztest, WST) [42] and State-Trait Anxiety Inventory (STAI) [43]. PD patients were clinically examined using the Unified Parkinson’s Disease Rating Scale (UPDRS) [44] and the Hoehn & Yahr Scale [45] in the OFF state. Disease specific cognitive deficits were evaluated using the Parkinson Neuropsychometric Dementia Assessment (PANDA) [46].

Group differences of normally distributed values (age, STAI-state, WST) were compared by two-sample t-tests, whereas group differences of values that were not normally distributed (distribution skewed, Shapiro-Wilk-Test: p < 0.01) were tested nonparametrically by the Mann-Whitney U test (BDI, EHI, MMSE). Statistical analysis was performed with SPSS Statistics 22 (IBM, USA) and values are presented as mean ± standard deviation. Statistical significance was considered at a threshold of p < 0.05.

### Pain and sensory threshold recordings

Individual warmth perception thresholds (WPT) and heat pain thresholds (HPT) were examined with the Medoc TSA-II (Medoc, Ramat. Yishai, Israel) thermal pain stimulation device (“ascending methods of limits” option; starting temperature 32°C, rise time 0.5°C/s). Warmth sensation was induced by a 9 cm^2^ Peltier thermode, centered on right volar forearm. For WPT, subjects signaled the first-time warmth sensation by a button press. For HPT, subjects signaled the first-time pain sensation. Subjects were familiarized with the procedure performing three test trials, the five following main trials were averaged to determine the individual WPT and HPT [47,48].

### FMRI paradigm

According to published implementations of laser heat pain stimulation in fMRI [49–51], a “Low Pain” laser stimulus with lower energy (LPS, E = 440 mJ) and a “High Pain” laser stimulus with higher energy (HPS, E = 640 mJ) were applied to right foot dorsum by a Thulium(Tm)-YAG-Laser (THEMIS, StarMedTec GmbH, Starnberg, Germany). After each stimulus, the application unit was moved about 1 cm within a 3 x 3 cm surface. First, 3 LPS and 3 HPS were applied outside the scanner to familiarize subjects with the pain stimulation. The perceived pain intensity of each test stimulus was evaluated on a 10 cm visual analogue scale (VAS, 0 cm “no pain”, 10 cm “most severe imaginable pain”). VAS values of the 3 LPS and 3 HPS were averaged as a measure of individually perceived pain intensity during application of test stimuli. During fMRI, subjects received 40 LPS in a first session, followed by 30 HPS in a second session. This approach was chosen to minimize uncertainty and associated expectation effects for upcoming painful stimuli. The interstimulus interval was randomized between 10 s to 20 s (mean LPS session 15.59 s, mean HPS session 15.43 s). Stimulus evaluation was not implemented in the fMRI paradigm to minimize cognitive influences on the imaging data.

### FMRI acquisition, preprocessing, and analysis

MRI was performed on a 3T scanner (Ingenia, Philips, Best, The Netherlands) equipped with an 8-channel SENSE head coil. T2*-weighted echo planar imaging (EPI) sequences were acquired (repetition time (TR) = 2595 ms, echo time (TE) = 35 ms, flip angle = 90°, field of view (FOV) = 230 x 230 x 147mm^3^, voxel size = 3.6 x 3.6 x 3.6 mm^3^, 41 axial slices in interleaved ascending mode). Scan duration was 11 min 7 s (LPS session) and 8 min 5 s (HPS session), resulting in 250 LPS and 180 HPS volumes per subject. This difference arose from piloting data which indicated lower skin conductance response (SCR) detection rates for LP compared to HP stimulation. In this data, an SCR could be measured in 80% of the applied LPS and in 100% of the applied HPS. Accordingly, LP stimuli number was adapted (40 low pain stimuli vs. 30 high pain stimuli). A high-resolution T1-weighted 3D-MPRAGE sequence (TI = 1300 ms, TR = 7.7 ms, TE = 3.9 ms, flip angle = 15°; FOV = 256 × 256 × 180 mm^3^, isotropic voxel resolution = 1 × 1 × 1 mm^3^, 180 slices, duration: 4 min 39 s) was acquired for anatomical reference and spatial normalization. The overall scanning duration including additional sequences (resting brain: 11 min 7 s, 3D-T2: 6 min 3 s, FLAIR: 5 min 31 s, and DTI: 8 min 38 s) was 55 min 10 s.

Preprocessing and analysis were performed with SPM8 (Wellcome Department of Imaging Neuroscience, London, UK) including slice-timing correction, realignment to the first volume, spatial normalization and smoothing (8 x 8 x 8 mm^3^). Exclusion criteria for imaging data were translational movements above the half-voxel resolution. However, no imaging data had to be excluded for movement artifacts. The first level design matrix consisted of the pain stimulation onsets convolved with the canonical hrf as implemented in SPM8 and six motion regressor derived from the realignment as nuisance factors. Both sessions (session 1: LPS, session 2: HPS) were in one model as separate sessions. The two contrast images contained the statistical effects of pain against baseline (LPS and HPS) and were used for further analysis at second level. Per group main pain effects of LPS and HPS were analyzed by use of a one-sample t-test. Between-group analysis was performed with a two-sample t-test for LPS and HPS condition separately. Statistical significance was considered for voxels exceeding a threshold of p < 0.05 (corrected for family wise errors at cluster level). Significant activations were localized using SPM Anatomy Toolbox Version 2.0 [52] and MRIcron (Version 6.6.2013, Chris Rorden, www.mricro.com).

### Behavioral data

Subjects were instructed to evaluate 60 laser-pain stimuli on a VAS. In total, 10 x 6 randomized laser heat pain stimuli with an energy of 440 mJ (LPS), 480 mJ, 520 mJ, 560 mJ, 600 mJ and 640 mJ (HPS) were applied to the right foot dorsum with an varying interstimulus interval (ISI) between 10 s to 20 s (mean ISI 16.1 s). During the ISI an evaluation of the pain stimuli followed using a VAS. VAS values were averaged for each of the six different pain stimuli. Group differences between the not normally distributed values of VAS (distribution skewed, Shapiro-Wilk-Test: p < 0.001) were investigated nonparametrically by the Mann-Whitney U test. Intra-group differences of VAS values were tested with the Friedman test plus post hoc testing. Linear correlations between target energy and VAS values were investigated by Spearman’s correlation coefficient.

### SCR data

During the behavioral assessment, we simultaneously acquired the SCR [53]. Ag/AgCl electrodes (BIOPAC Systems, Inc.) were attached to the palmar index and the middle finger of the left hand. Data was acquired with the data acquisition unit MP150 (BIOPAC Systems, Inc.). Electrodermal Activity (EDA) was recorded in DC mode (gain 5 μmho/V, low pass filter 1 Hz, sampling rate 100 Hz) and processed with AcqKnowledge Version 4.1 (BIOPAC Systems, Inc.) using the “Event-related EDA Analysis” option (high-pass filter of 0.05 Hz). SCR amplitudes were defined as the difference between maximum and baseline responses within a time window of 1–4 s after stimulus onset and rise times of 1–3 s [54]. Data was transformed logarithmically. SCR magnitudes were estimated for each of the six pain stimuli and standardized by z-transformation.

Group differences concerning standardized SCR magnitudes were compared by two-sample t-tests. Intra-group differences of SCR-magnitudes were tested by a one-way analysis of variance (ANOVA) with repeated measures and Bonferroni corrected post hoc tests. Linear correlations between target energy and SCR magnitude were investigated by Pearson’s correlation coefficient.

One PD patient and one control subject were excluded from EDA analysis due to artificial EDA data.

## Results

### Clinical and neuropsychological characterization

None of the participants showed results indicating psychiatric diseases (MINI). MMSE results of both groups showed no cognitive deficits (score ≥ 27 points) or significant group differences regarding cognitive capacity (PD: 29.38 ± 0.96 points; HC: 29.77 ± 0.44 points; U = 68.50, p = 0.418).

Significant group differences were observed for BDI-II, STAI-state and WST. PD had significantly higher scores in BDI (PD: 5.62 ± 3.45 points, range 0–11 points; HC: 1.46 ± 1.85 points, range 0–6 points; U = 143.5, p = 0.002), STAI-state (PD: 35.85 ± 8.09 points; HC: 26.85 ± 4.36 points, t(24) = 3.53, p = 0.002), and significantly lower scores in WST (PD: 103.15 ± 11.10 points; HC: 112.46 ± 11.04 points; t(24) = -2.14, p = 0.042).

The PANDA test yielded consistent results, both for cognition and mood (Table 1). Although one PD patient showed a score of 14 points at the threshold level (PANDA cognition part), the subject was not excluded due to a normal MMSE score. To consider possible effects of increased situational anxiety and different mood states, we performed additional fMRI models with BDI and STAI-state scores as covariates at the second level.

### Warmth perception and thermal heat pain threshold

Temperatures of WPT and HPT did not differ significantly between PD and HC (WPT_PD_: 33.83 ± 1.02°C vs. WPT_HC_: 34.01 ± 1.65°C, t(24) = -0.33, p = 0.741; HPT_PD_: 44.71 ± 1.91°C vs. HPT_HC_: 45.97 ± 1.40°C, t(24) = -1.93, p = 0.066).

### Evaluation of laser stimuli with different target energies

The evaluation of the pain stimuli with six different target energies (each applied 10 times) didn’t result in significant group differences of VAS values (Table 2). In both groups, post hoc tests showed that HPS was evaluated by significant higher VAS values than LPS (PD: p < 0.001; HC: p < 0.001). Target energy and VAS values were significantly correlated (PD: rs = 0.60, p < 0.001; HC: rs = 0.63, p < 0.001). The correlation between target energy and VAS values was not significantly different between PD and HC (p = 0.795).

**Table 2:** Behavioral data evoked by laser-induced pain stimulation.

	VAS (cm)			SCR magnitude (z score)		
target energy (mJ)	PD	HC	group effect	PD	HC	group effect
440 (= LPS)	0.41 ± 0.84	0.34 ± 0.33	NS	-0.88 ± 0.30	-0.64 ± 0.92	NS
480	0.68 ± 0.95	0.58 ± 0.51	NS	-0.50 ± 0.85	-0.60 ± 0.49	NS
520	1.12 ± 1.30	0.94 ± 0.82	NS	-0.49 ± 0.53	-0.42 ± 0.61	NS
560	1.68 ± 1.68	1.51 ± 1.37	NS	0.06 ± 0.58	0.21 ± 0.79	NS
600	2.27 ± 1.89	2.10 ± 1.82	NS	0.55 ± 0.61	0.50 ± 0.67	NS
640 (= HPS)	2.67 ± 1.97	2.50 ± 1.96	NS	1.25 ± 0.49	0.95 ± 0.76	NS
within group effect	χ^2^(5) = 61.84, **p < 0.001**	χ^2^(5) = 49.64, **p < 0.001**		F(3,28) = 18.42, **p < 0.001**	F(5,55) = 8.31, **p < 0.001**	

Data was collected immediately after the fMRI investigation.

HC: control subjects, HPS: high pain laser stimulus (E = 600 mJ), LPS: low pain laser stimulus (E = 440 mJ), PD: patients with early-stage Parkinson’s disease (OFF state), SCR: skin conductance response, VAS: visual analogue scale.

### Electrodermal responses

Electrodermal measurements did not result in significant group differences of standardized SCR magnitudes (Table 2). The different target energies yielded significantly different standardized SCR magnitudes (Table 2). In both groups, post hoc tests showed that HPS led to significant higher standardized SCR magnitudes than LPS (PD: p < 0.001; HC: p < 0.025). Target energy and standardized SCR magnitudes were significantly correlated (PD: r = 0.77, p < 0.001; HC: r = 0.64, p < 0.001) and showed a linear relationship (PD: r^2^ = 0.59, y = -5.53 + 0.01x; HC: r^2^ = 0.41, y = -4.59 + 8.5·10^-3^x). The correlation between target energy and SCR magnitudes was not significantly different between PD and HC (p = 0.125).

### FMRI data

#### Central pain activation in PD patients (OFF state) and in healthy controls

**Fig 2:**
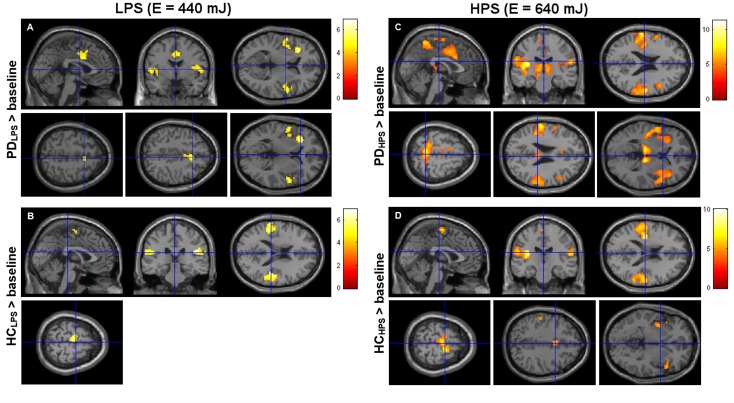
Central activation for the LPS and HPS pain condition in early-stage PD and healthy controls. Central activation (p < 0.05, FWE cluster corrected) in early-stage PD patients (A, C) and healthy controls (B, D) for the LPS (A, B) and HPS (C, D) condition. For activated regions see also Table 3 and Table 4. HC: healthy controls, HPS: high pain laser stimulus with higher target energy (E = 600 mJ), LPS: low pain laser stimulus with lower target energy (E = 440 mJ), PD: patients with early-stage Parkinson’s disease in OFF state.

For LPS, PD patients showed significant activation (p < 0.05, FWE cluster corrected) in ipsilateral supplementary motor cortex (SMA), bilateral in parietal operculum/secondary somatosensory cortex (S2) (contralateral: OP1-4; ipsilateral: OP4) (LPS_PD_ > baseline), mesial cingulate cortex (MCC), and contralateral insula (Ig1, Ig2) (Fig 2A, Table 3). For LPS, control subjects showed significant activations (p < 0.05, FWE cluster corrected) in parietal operculum/S2 (contralateral: OP1, OP3, OP4; ipsilateral: OP1, OP3), inferior parietal cortex (contralateral: PFcm, PFop; ipsilateral: PFcm, PFop), and in primarily contralateral SMA (LPS_HC_ > baseline) (Fig 2B, Table 4).

**Table 3:** Main effects of laser pain stimulation in PD patients.

Cytoarchitectonic region	Side	K	T	Z	x	y	z
**LPS**_**PD**_ **> baseline**							
Mesial cingulate cortex	R	207	6.06	4.02	6	17	46
Mesial cingulate cortex	R		5.36	3.76	6	8	37
Supplementary motor area	R		5.03	3.62	6	8	55
Insula lobe (OP2)	L	116	5.10	3.65	-33	-22	16
Rolandic operculum (OP4)	L		5.07	3.64	-51	-1	4
Insula lobe	L		4.50	3.38	-39	5	7
Rolandic operculum (OP4)	R	99	5.07	3.64	51	-1	7
Inferior frontal gyrus (p. opercularis)	R		4.71	3.48	51	11	13
Insula lobe, OP3	R		4.54	3.40	36	-1	10
Insula lobe	L	87	6.91	4.31	-30	26	4
*Rolandic operculum*	R	73	5.32	3.74	45	-19	22
*Rolandic operculum (OP1)*	R		4.67	3.46	63	-19	16
*Supramarginal gyrus*, *PFop (IPL)*	R		4.49	3.37	51	-28	25
*Postcentral gyrus*, *PFop (IPL)*	L	59	4.73	3.49	-63	-22	19
*Supramarginal gyrus*, *PFop (IPL)*	L		4.52	3.39	-60	-28	28
*Superior temporal gyrus*, *PFcm (IPL)*	L		4.30	3.28	-54	-34	13
**HPS**_**PD**_ **> baseline**							
Insula lobe, OP2	L	1667	11.31	5.34	-33	-19	16
Thalamus, parietal	R		9.04	4.88	12	-25	4
OP1	L		8.76	4.82	-45	-19	10
Insula, Id1	R	837	9.04	4.88	42	-1	-17
Rolandic operculum, Pfop (IPL)	R		8.36	4.72	57	-16	19
Insula lobe, Ig2	R		6.96	4.33	39	-13	10
Mesial cingulate cortex	L	522	7.24	4.41	-6	8	40
Mesial cingulate cortex	R		6.47	4.17	3	8	43
Anterior cingulate cortex	L		6.46	4.17	-3	17	31
Precuneus, 5m (SPL)	L	392	7.42	4.46	-6	-49	61
Precuneus, 5l (SPL)	R		7.32	4.43	9	-52	64
Superior parietal lobule, 5l (SPL)	L		7.21	4.40	-18	-43	64
Posterior cingulate cortex	R	57	6.19	4.07	6	-31	25

Maxima of activation clusters for the LPS and HPS condition in patients with early-stage Parkinson’s disease (PD, OFF state). Statistical threshold: p < 0.05, FWE cluster corrected; italic: trend activation at p < 0.001, uncorrected (extent threshold 59 vx).

HPS: high pain laser stimulus (target energy E = 600 mJ), IPL: inferior parietal lobule, K: cluster size, L: left, LPS: low pain laser stimulus (target energy E = 440 mJ), OP: parietal operculum, R: right, SPL: superior parietal lobule, T: T values, X/Y/Z: coordinates in MNI space, Z: Z scores.

**Table 4:** Main effects of laser pain stimulation in healthy control subjects.

Cytoarchitectonic region	Side	K	T	Z	x	y	z
**LPS**_**HC**_ **> baseline**							
Superior temporal gyrus, PF (IPL)	L	134	6.21	4.08	-63	-31	19
Superior temporal gyrus, OP1	L		5.89	3.97	-48	-31	19
Postcentral gyrus, OP1	L		5.02	3.62	-51	-19	22
Rolandic operculum, PFop (IPL)	R	113	6.94	4.32	45	-28	22
Superior temporal gyrus, PFcm (IPL)	R		5.11	3.65	54	-37	19
Supramarginal gyrus, PFt (IPL)	R		4.67	3.46	63	-19	25
Superior frontal gyrus	L	97	6.21	4.08	-21	-10	61
Paracentral lobule	L		6.01	4.01	-12	-16	70
**HPS**_**HC**_ **> baseline**							
Insula lobe, OP2	L	356	10.05	5.10	-33	-19	19
Postcentral gyrus, PFt (IPL)	L		6.61	4.21	-57	-22	31
Superior temporal gyrus, OP1	L		6.42	4.15	-54	-28	19
Rolandic operculum, OP1	R	202	6.97	4.33	57	-19	16
Rolandic operculum, PFcm	R		6.16	4.06	45	-31	22
Superior temporal gyrus, PFm	R		6.14	4.05	57	-43	19
Supplementary motor area	R	169	8.41	4.73	15	-7	67
Supplementary motor area	L		6.88	4.30	0	-13	67
Supplementary motor area	L		5.15	3.67	-15	-7	67
Rolandic operculum	R	149	5.86	3.95	51	2	7
Inferior frontal gyrus (p. Opercularis)	R		5.52	3.82	51	17	1
Insula lobe	R		4.53	3.39	36	11	7
Temporal pole	L	114	6.43	4.16	-39	2	-14
Insula lobe	L		6.12	4.05	-39	-1	-5
Insula lobe	L		4.82	3.53	-33	8	7
Mesial cingulate cortex	L	66	5.25	3.71	0	5	40
Mesial cingulate cortex	L		4.70	3.47	-6	2	46

Maxima of activation clusters for the LPS and HPS condition in healthy control subjects (HC). Statistical threshold: p < 0.05, FWE cluster corrected.

HPS: high pain laser stimulus with higher target energy (E = 600 mJ), IPL: inferior parietal lobule, K: cluster size, L: left, LPS: low pain laser stimulus with lower target energy (E = 440 mJ), OP: parietal operculum, R: right, T: T values, X/Y/Z: coordinates in MNI space, Z: Z scores.

For HPS, PD patients showed significant activations (p < 0.05, FWE cluster corrected) in parietal inferior lobule (PFcm, PFm, PFop, PFt), parietal operculum/S2 (OP1-4), SMA, mesial cingulate cortex, and in the insula (contralateral: Ig2, Id; ipsilateral: Ig1, Ig2, Id1) (HPS_PD_ > baseline). Additional bilateral activation was observed in superior parietal lobule/precuneus (5m, 5l, 5Ci), anterior and posterior cingulate cortex (ACC, PCC), and in thalamus (Fig 2C, Table 3). For HPS, control subjects showed significant bilateral activation (p < 0.05, FWE cluster corrected) in parietal operculum/S2 (contralateral: OP1-4; ipsilateral: OP1, OP4), inferior parietal cortex (contralateral: PFcm, PFop, PFt; ipsilateral: PFcm, PFm, PFop, PFt), SMA, and insula (Ig1, Ig2) (Fig 2D, Table 4).

#### Between group comparison: PD patients (OFF state) vs. healthy controls

**Fig 3:**
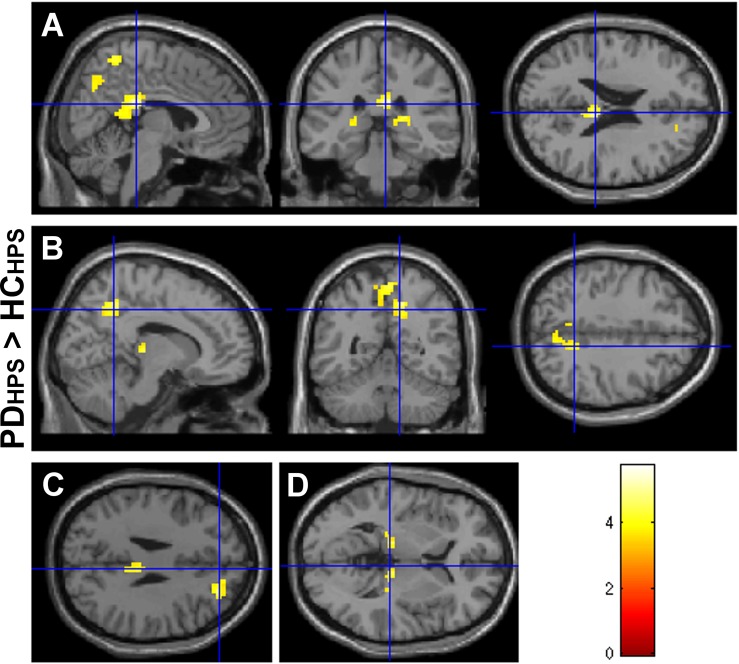
Increased central activation in early-staged PD patients vs. healthy controls for the HPS condition. Increased central activation (p < 0.05, FWE cluster corrected) for the high laser pain condition (HPS, target energy E = 600 mJ) in patients with early-staged Parkinson’s disease (PD, OFF state) vs. healthy control subjects (HC) in posterior cingulate cortex (A) and precuneus (B). Trend activation (p < 0.001, uncorrected) in ipsilateral medial frontal gyrus (C) and bilateral thalamus (D).

For LPS, the between group comparison of activation in PD patients and healthy controls (contrasts: LPS_PD_ > LPS_HC_, LPS_HC_ > LPS_PD_) did not reveal any significant differences. On the contrary, the between group comparison for HPS (contrasts: HPS_PD_ > HPS_HC_, HPS_HC_ > HPS_PD_) showed increased activations (p < 0.05, FWE cluster corrected) in precuneus and PCC in PD patients (Fig 3, Table 5). For visualization purposes and to demonstrate the full extent of the activation differences, the statistical threshold was reduced to p < 0.001, uncorrected, showing also a tendency for increased activations in ipsilateral medial frontal gyrus and thalamus bilaterally. Hence, relatively increased activations in PD were localized in regions of the default mode network (DMN), containing medial prefrontal cortex (mPFC), PCC, precuneus, lateral parietal cortex and medial temporal cortex [55,56]. To test whether these effects were driven by BDI or STAI-state values, a regression analysis was performed in the PD group (p < 0.001, uncorrected). This analysis found no indication that the precuneus/PCC activation is driven by the covariates BDI plus STAI-state.

**Table 5:** Between group effects (PD vs. HC) for the HPS condition.

Cytoarchitectonic region	Side	K	T	Z	x	y	z
Between group effects HPS_PD_ > HPS_HC_							
Precuneus	R	97	4.86	4.01	12	-52	46
Precuneus	R		3.94	3.43	9	-70	37
Precuneus, 5m (SPL)	R	92	4.67	3.90	3	-49	61
Precuneus	L		3.78	3.32	-6	-55	43
5Ci (SPL)	L		3.76	3.30	-15	-46	46
Posterior cingulate cortex	R	91	5.72	4.50	6	-31	22
Precuneus	R		4.03	3.49	6	-46	13
*Medial frontal gyrus*	R	49	4.65	3.89	27	41	28
*Thalamus*, *parietal*	L	45	4.40	3.73	-15	-25	4
*Thalamus*, *temporal*	L		4.19	3.59	-9	-25	13
*Thalamus*, *parietal*	R	38	4.79	3.97	21	-28	7

Maxima of activation clusters for the HPS condition in patients with early-staged Parkinson’s disease (PD, OFF state) vs. healthy control subjects (HC). Statistical threshold: p < 0.05, FWE cluster corrected; italic: trend activation at p < 0.001, uncorrected (extent threshold 38 vx). HC: control subjects, HPS: high pain laser stimulus with higher target energy (E = 600 mJ), K: cluster size, L: left, LPS: low pain laser stimulus with lower target energy (E = 440 mJ), R: right, SPL: superior parietal lobule, T: T values, X/Y/Z: coordinates in MNI space, Z: Z scores.

## Discussion

This is the first study investigating central pain perception in PD using a multimodal experimental approach, notably at behavioral, autonomic, and imaging levels. No significant differences in WPT, HPT, EDA and subjective pain ratings were found between early-stage PD patients and controls, and erfMRI revealed a generally comparable activation pattern in brain areas belonging to the central pain matrix. Relatively reduced deactivation was found in PD in posterior regions of the DMN, notably the precuneus and the posterior cingulate cortex. In the following the results are discussed at the peripheral and central levels.

### Peripheral pain perception in PD

When investigating pain discrimination in PD, careful clinical assessments are essential to account for the elevated prevalence of pain [16,57,58], and the association of chronic pain, depression and anxiety disorders in PD [59–63]. Therefore, we enrolled exclusively PD patients without chronic pain or psychiatric diseases, minimizing secondary effects on pain perception. Although our PD patients showed higher BDI scores and state anxiety values, we didn’t observe significant group differences concerning warmth perception thresholds, heat pain thresholds, laser-induced pain perception or electrodermal measurements. Thus, we assume a negligible effect of these parameters on sensory discriminative levels of pain perception.

Compared to HC, our behavioral investigations revealed unaltered warmth and thermal heat pain thresholds, and comparable laser-induced cutaneous pain perception in early-stage PD patients tested in the OFF state. We thus assume comparable peripheral sensory and nociceptive transmission, a claim further strengthened by electrophysiological exclusion of peripheral neuropathy. Thus, while our results support former reports of unaltered warmth perception thresholds in PD [64], we couldn’t confirm previous work reporting decreased pain thresholds [35,64–67] and, thus, found no support for a modified sensory discriminative pain perception in early-stage PD. Indeed, the literature on altered sensory discriminative pain perception is highly inconsistent [36,68,69]. This might be explained to some extent by patient selection differences (e.g. disease stage, presence of chronic pain, pharmacological treatment, etc.). For instance, associations between decreased pain thresholds and severity of motor symptoms indicate that pain in PD might be triggered by rigidity or bradykinesia [66]. In addition, neuropathological deficits at the level of peripheral nerves could have affected pain perception, as the presence of peripheral sensory dysfunction wasn’t systematically ruled out in all of the mentioned studies. Finally, variable test methods including a wide range of stimulus modalities (e.g. thermic, electric, or laser-induced), stimulus localizations (e.g. upper vs. lower extremities) and stimulus durations (< 1 ms to 90 s) might explain the mentioned inconsistencies in the literature.

Our electrodermal measurements during laser-induced pain also didn’t reveal any significant group differences, suggesting unaltered autonomic response to nociception in early-stage PD patients.

### Central pain activation in PD

Laser-induced nociceptive stimulation induced a pain-specific activation pattern encompassing regions of the central pain matrix (CPM) [70–74], including secondary somatosensory cortex (S2), insula, cingulate cortex and thalamus, both in PD patients and healthy controls. Besides group difference found in DMN regions, no significant group differences were observed within regions of the CPM, either in HPS or LPS pain conditions. This suggests a comparable recruitment of areas involved in sensory discriminative or affective nociceptive processing like SII [50,73,75,76] and the insula [70,72,73,77] in early-stage PD patients and controls. This finding is contrary to our study hypothesis, influenced by the hitherto only imaging study examining central pain processing in PD [35]. Brefel-Courbon et al. reported increased activation in PD in regions involved in affective nociceptive processing (i.e. ipsilateral posterior insula, prefrontal cortex, contralateral ACC). Although these PD patients were in a more advanced clinical stage (Hoehn and Yahr stage 2.2 vs.1.27), the validity and reproducibility of these results at a very liberal statistical threshold has to be questioned [35]. Moreover, no systematic screening for peripheral sensory dysfunction was performed [36], or for neuropsychological factors affecting pain sensation (e.g. depressive symptoms, situational anxiety).

### Default mode network in PD

Relative increases of HPS-induced activation were observed in precuneus and PCC in the PD group. The changes reflect decreased deactivation in the PD group, as compared to HC and reinforce former imaging data showing abnormal processing of external stimuli and increased activity (resp. decreased deactivation) of the DMN in PD patients [78,79]. To our knowledge, this is the first study showing DMN dysfunction in PD patients during nociceptive processing. As both covariates did not correlate with the precuneus and PCC activation in the PD cohort, it is rather unlikely that the observed DMN effects are driven by mood or anxiety levels. Alterations of DMN activity and connectivity have also been described in states of altered cognitive processing, both in physiological aging and neurodegenerative diseases [80,81]. DMN dysfunction has been reported also in neuropsychiatric disorders like autism, schizophrenia, Alzheimer’s disease and depression [82,83].

### Limitations

A limiting factor of our study is the small sample size of subjects which is mainly a consequence of adherence to strict exclusion / inclusion criteria, notably using electrophysiological testing to rule out individuals with evidence of peripheral neuropathy which may influence conductance of nociceptive stimuli and affect central pain-related activation patterns. We chose these criteria for the benefit of high-quality data in homogeneous collectives with highly diminished confounding effects on pain perception.

## Conclusion

Early-stage PD patients show abnormalities of DMN function during nociceptive processing, extending previous findings in other behavioral domains. Experimental correlates of nociceptive processing, as tested at behavioral, autonomic, imaging levels, revealed no genuine pain-specific processing abnormality in early-stage PD, after exclusion of peripheral neuropathy using electrophysiological screening. Applying similar rigorous methodological approaches, future studies are now required to examine pain processing in more advanced stages of PD.
